# Evaluation of an ex vivo fibrogenesis model using human lung slices prepared from small tissues

**DOI:** 10.1186/s40001-023-01104-8

**Published:** 2023-03-30

**Authors:** Ying Sun, Pengyu Jing, Helina Gan, Xuejiao Wang, Ximing Zhu, Jiangjiang Fan, Haichao Li, Zhipei Zhang, James Chi Jen Lin, Zhongping Gu

**Affiliations:** 1 Department of Thoracic Surgery, The Second Affiliated Hospital, Air Force Medical University, Xi’an, 710038 China; 2 Fibroscience LLC, 8037 Glengarriff Rd., Clemmons, NC 27012 USA

**Keywords:** Human lung slice, Cadmium chloride, TGF-β1, Fibroblast foci, MMP1

## Abstract

**Background:**

In recent years, there have been breakthroughs in the preclinical research of respiratory diseases, such as organoids and organ tissue chip models, but they still cannot provide insight into human respiratory diseases well. Human lung slices model provides a promising in vitro model for the study of respiratory diseases because of its preservation of lung structure and major cell types.

**Methods:**

Human lung slices were manually prepared from small pieces of lung tissues obtained from lung cancer patients subjected to lung surgery. To evaluate the suitability of this model for lung fibrosis research, lung slices were treated with CdCl_2_ (30 μM), TGF-β1 (1 ng/ml) or CdCl_2_ plus TGF-β1 for 3 days followed by toxicity assessment, gene expression analysis and histopathological observations.

**Results:**

CdCl_2_ treatment resulted in a concentration-dependent toxicity profile evidenced by MTT assay as well as histopathological observations. In comparison with the untreated group, CdCl_2_ and TGF-β1 significantly induces MMP2 and MMP9 gene expression but not MMP1. Interestingly, CdCl_2_ plus TGF-β1 significantly induces the expression of MMP1 but not MMP2, MMP7 or MMP9. Microscopic observations reveal the pathogenesis of interstitial lung fibrosis in the lung slices of all groups; however, CdCl_2_ plus TGF-β1 treatment leads to a greater alveolar septa thickness and the formation of fibroblast foci-like pathological features. The lung slice model is in short of blood supply and the inflammatory/immune-responses are considered minimal.

**Conclusions:**

The results are in favor of the hypothesis that idiopathic pulmonary fibrosis (IPF) is mediated by tissue damage and abnormal repair. Induction of MMP1 gene expression and fibroblast foci-like pathogenesis suggest that this model might represent an early stage of IPF.

## Background

Lung epithelium injuries are normally repaired immediately through inflammatory responses involving cytokine releasing, inflammatory cell recruitment, coagulation activation and followed by the regeneration of epithelium through progenitor cell proliferation and differentiation [1]. Environmental toxicant insult is considered associated with COPD [2], pulmonary fibrosis [3] and asthma [4]; recurrent toxicant exposure followed by impaired tissue repair might underline the mechanism of their pathogenesis. Very often, animal models are used to monitor the pathogenesis from early lung injury to chronic diseases [5–7] knowing the differential responses might exist between species upon xenobiotic exposures [8]. As good examples, chronic exposure of cadmium is highly correlated with emphysema [9]; in addition, repeated cadmium exposure through inhalation resulted in fibrosis and emphysema in animals [10]. However, translational interpretation of such results to human exposure may encounter difficulties due to the differential tolerant levels upon cadmium treatment [11]. Hence, 3D experimental models using human lung cells or tissues are preferred and suggested for translational research [12].

Among these 3D models, ex vivo human lung slice has shown its validity in studying parenchymal toxicity, inflammation and pathological alterations [13–15] as it maintains the features of cell–cell and cell–matrix interactions that are critical in the pathogenic process. Preparation of human lung slices usually requires a whole lung lobe and human lung slices are not regularly used as animal lung slices due to the limited access to human lung source. Therefore, this study intends to facilitate the use of human lung slices by developing a method that only requires small pieces of tissues for lung slice preparation. To evaluate this lung slice preparation method, human lung slices were subjected to cadmium or cadmium plus TGF-β1 treatment. The results of the induced toxicity, metalloproteinase (MMP) gene expression profiles and ex vivo fibrogenesis in human lung slices were compared with the published data.

Maintenance of lung slice in the air–liquid interphase (ALI) is considered closer to in vivo conditions compared to immersion culture; it is noteworthy that a previous published ALI culture set up is applied in this study [16, 17]. Cadmium is chosen for two reasons. First, in vitro, ex vivo and in vivo approaches have been taken to address the cadmium-associated adverse pulmonary effects [18–20]. Second, CdCl_2_ plus TGF-β1 treatment in the rat lung slices was shown to induce fibrous alveoli [16]. Current results strongly suggest that the lung slices prepared from the proposed method is valid as a translation model for CdCl_2_-induced toxicity; and further suggest it a useful model linking tissue damage to the early stage lung fibrosis.

## Methods

### Lung specimens and ethical statement

Lung tissue blocks were obtained from the non-smoking patients undergoing surgical cancer treatment. Written informed consent was obtained from all subjects and the study was approved by the Regional Ethics Committee for Clinical Research of the Air Force Medical University.

### Media for lung slice preparation and culture

Basal medium consists of RPMI 1640 (Gibco, NewYork, USA), penicillin/streptomycin (10 UI and 10 µg/ml; Hyclone, Los Angeles, USA), retinyl acetate (0.1 µg/ml; Sigma Aldrich, Saint Louis, USA), bovine Insulin (1.0 µg/ml; Sigma Aldrich, Saint Louis, USA), hydrocortisone (1.0 µg/ml; Sigma Aldrich, Saint Louis, USA), Nystatin (1.0 µg/ml; Sigma Aldrich, Saint Louis, USA), L-Glutamine (2 mM; Sigma Aldrich, Saint Louis, USA) and fungizone (0.625 µg/ml; Sigma Aldrich, Saint Louis, USA). Basal medium is prepared and stored at 4 °C, then Fungizone and Nystatin were added prior to use. Agarose infiltration medium was prepared by freshly mixing basal medium and low melting agarose gel at a 1–1 ratio to achieve the target agarose concentration of 1.2%; low melting agarose gel is prepared in PBS and melted by autoclave. AIM was kept pre-heated in 45 °C water bath until use. Complete medium consists of BM plus 0.5% Fetal Calf Serum (Excell Bio, Uruguay), stored at 4 °C no more than 10 days.

### Lung slice preparation and culture

Human lung tissue blocks (roughly 1 cm^3^ and 5 cm away from the tumor site) obtained from surgery were immediately placed in the BM and kept at 4 °C no longer than 3 h until agarose infiltration. For agarose infiltration, four lung blocks were put into a 20 ml syringe, 15 ml of agarose infiltration medium (temperature measured no higher than 38 °C) was added to the syringe and followed by slow manual forcing with syringe plunger. Such gradual pressure was given within 1-min duration so as to allow losing of 5 ml medium through the filter at the lower part of the apparatus, then the pressure remained for 1 min without further forcing. Afterward, a 30 s cooling process using ice cold paper towel to warp around the syringe was immediately applied, while the same pressure was held. The whole apparatus was moved to refrigerator for 30 min to ensure the complete solidification of agarose. Agarose filled lung tissue blocks were then removed by carefully disassembling the apparatus under the aseptic condition. Lung slices of 0.5 cm^2^ and 1.0–1.5 mm in thickness were prepared by manually slicing. Rat lung slices were prepared by the same method described previously [16] with only one modification that 0.6% low melting agarose in basal medium was applied to inflate the rat lungs through trachea infiltration. 

Lung slices were rinsed in the complete medium before they were placed onto a culture device. Lung slices were maintained with complete medium in the air/liquid interphase at 37 °C in a humidified incubator supplemented with 5% CO_2_.

### Treatment to the lung slices

Right after preparation, lung slices were cultured with complete medium for a 48-h acclimation before treatment. For treatment, lung slices were refreshed with complete medium (untreated group), or complete medium containing CdCl_2_ (30 μM, Sigma Aldrich, Saint Louis, USA), TGF-β1 (1 ng/ml, Proteintech, Chicago, USA) or CdCl_2_ plus TGF-β1 and they were retained at the same condition for 3 days without changing medium. At the end of treatment, lung slices were harvested for toxicity, gene expression or histology evaluation. For the cultures further maintained after treatment, media was refreshed every other day.

### Toxicity and histology assessments

MTT assay was applied for toxicity assessment using the method previously described [21, 22]. Specifically, lung slices were immersed in an MTT-formazan solution (75 μg/ml) and incubated at 37 °C for 40 min. Formazan was extracted in isopropanol and the optical density was measured at 570 nm. The OD values were normalized by the lung slice’s total protein content measured by the BCA method. For histology assessment, lung slices were fixed with 10% buffered formalin at the end of post exposure culturing period. Paraffin-embedded lung slices were prepared, sectioned (5 µm) and processed for hematoxylin/eosin staining or trichrome stain.

### Gene expression

Lung slices were harvested and stored at − 80 °C until RNA extraction. They were thawed at room temperature and homogenized in Roche TriPure Isolation Reagent; after centrifugation, the aqueous phase was collected and further spin column RNA purification was performed according to manufacturer’s instructions. First-strand cDNA was synthesized using QIAGEN RT^2^ First Strand kit. SYBR Green real-time PCR analysis was conducted using commercial primer sets (QIAGEN) and reagents (Roche faststart universal SYBRGreen I Master) in a Mx3005P machine (Stratagene). Dual house-keeping genes, ribosomal protein (RPLP0) and β-2 microglobulin (B2M) were used for normalization. Acceptable PCR runs were verified by melting curve.

### Statistical analysis

Target gene expressions in the treated group compared with untreated group (fold change) were calculated by 2^−∆∆CT^ method; one sample *T* test on the fold change was used to evaluate the difference against the untreated group. Results are presented as the means ± SEM and data were collected from at least 3 independent experiments, where lung slices were prepared from a single subject in each experiment.

## Results

**Fig. 1:**
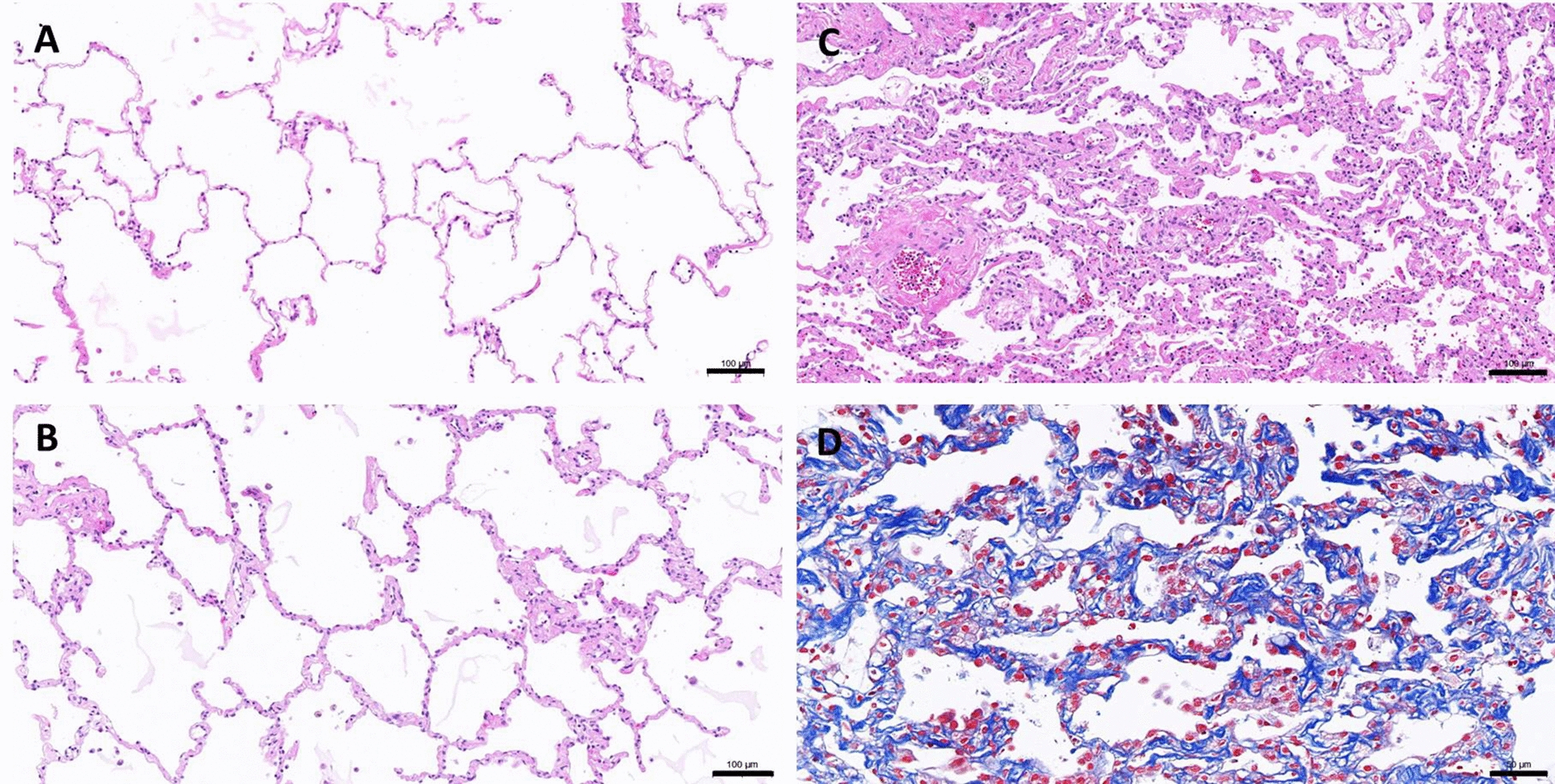
Microscopic observations of the untreated lung slices on the day of preparation (**a**, day 0), lung slices harvested following 2 days of acclimation (**b**, day 2) or harvested 3 days after acclimation period (**c**, day 5). Parafilm sections were subjected to H/E staining (**a**–**c**), where bars indicate 100 μm or Masson’s TriChrome staining (**d**), where bar indicates 50 μm

To evaluate the impact of physical stress derived from lung tissue preparation to the integrity of alveolar feature, lung slices were subjected to histological observation on the day of preparation (day 0), 2 days after acclimation and (day 2) and on the 5th day after preparation (day 5). Increased thickness of alveolar septa was clearly observed overtime (Fig. 1a–c); on day 5, the diffuse alveolar wall fibrosis was further evidenced by Masson’s trichrome stain (Fig. 1d).

**Fig. 2:**
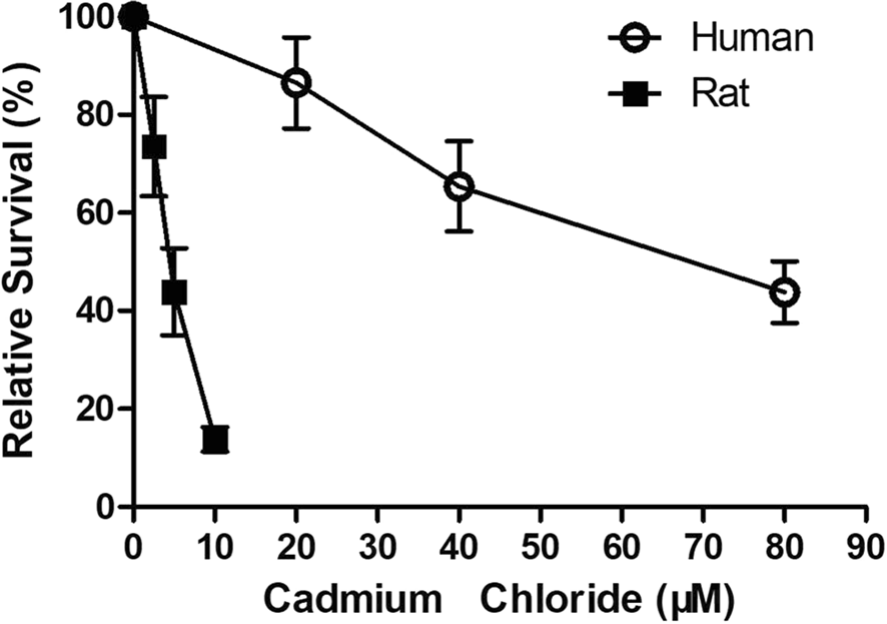
CdCl_2_-induced toxicity to the lung slices. Human (empty circle) or rat (solid square) lung slices were treated with CdCl_2_ at day 2 for 3 days and harvested at day 5 followed by MTT assay; indicated toxicity is a relative value in comparison with the untreated lung slices

**Fig. 3:**
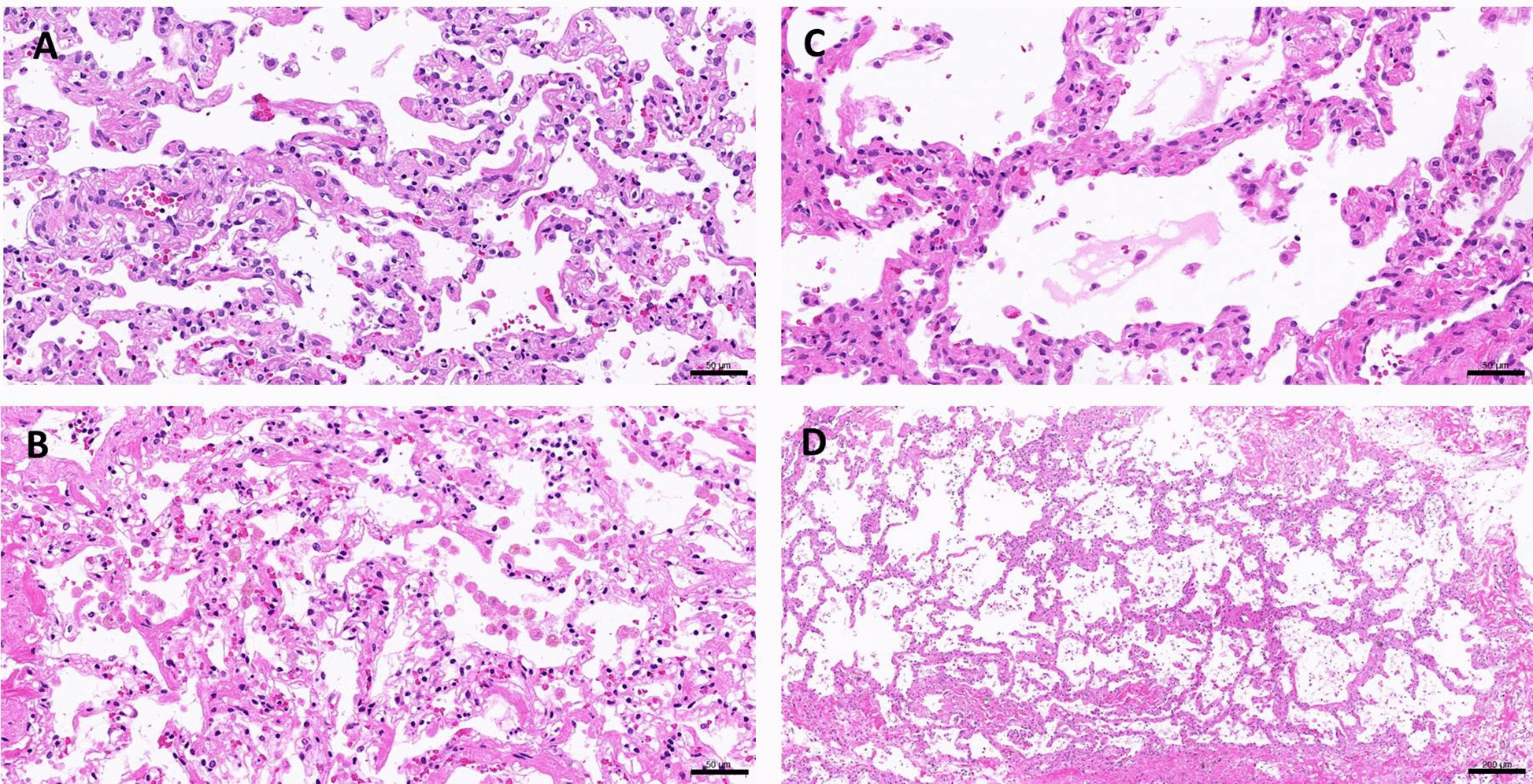
Microscopic observations (H/E staining) of the lung slices harvested on day 5 following a 3-day CdCl_2_ treatment with a concentration of 0, 20, 40 or 80 μM (**a**–**d,** respectively). Bars indicates 50 (**a**–**c**) and 200 μm (**d**)

The capability of tissue repair from damage in the cultured lung slices was examined by challenging them with cadmium chloride for 3 days. Following a 3-day treatment, clear dose–response toxicity profiles were observed both in rat and human lung slices (Fig. 2). Interestingly, human lung slices showed a stronger resistance against cadmium challenge in comparison with rat lung slices. Such cytotoxic effect was accompanied by the loss of lung tissue integrity that enlarged alveolar space as well as further thickened alveolar septa were observed in the lung slices treated with CdCl_2_ at 40 and 80 μM (Fig. 3c, d). It is estimated, from this toxicity profile, that a 3-day treatment with CdCl_2_ at 30 μM will generate a 25% relative cytotoxicity in comparison with the untreated human lung slices. Hence, tissue repair capability was evaluated by examining the expression of some extra cellular matrix (EMC)-associated genes in the lung slices treated with CdCl_2_ at this concentration. Results showed a tendency of increased expression of all 6 genes listed in Table 1; however, only MMP2 and MMP9 gene expression were statistically significant following CdCl_2_ exposure.

**Table 1:** Collagen metabolism-associated gene expression (fold change)

	Col 1a1		α-SMA		MMP 1		MMP 2		MMP 7		MMP 9	
	Mean	SEM	Mean	SEM	Mean	SEM	Mean	SEM	Mean	SEM	Mean	SEM
Cd	3.59	2.62	5.39	3.90	2.41	1.59	3.69*	0.88	6.42	4.03	3.22*	0.92
TGF-β1	2.20	0.97	1.02	0.01	1.57	0.99	2.15**	0.15	1.54	1.16	4.91**	1.57
Cd + TGF-β1	1.45	0.75	1.82	0.59	4.50	3.02	3.54	1.48	2.61	1.69	2.66	1.26

The Indicated fold change of gene expression is a relative value in comparison with the untreated group, value in each data point was collected from at least 3 independent experiments. Following the statistical analysis, a *P* values were obtained, where * and ** indicate a *P* value of < 0.05 and < 0.01, respectively

Since thickened alveolar wall, an indication of fibrosis, can be observed in the untreated and CdCl_2_-treated lung slices, we further examined whether advanced fibrous response can be triggered by TGF-β1, a key cytokine accountable for fibrogenesis. After 2 days of acclimation period, lung slices were maintained 3 further days with basal medium, the medium containing 1 ng/ml TGF-β1 or the medium containing CdCl_2_ (30 μM) plus TGF-β1. The lung slices were then harvested for gene expression. Results in Table 1 showed that, in comparison with the untreated group, TGF-β1-treated group seemed to exhibit higher expression rate of all genes except α-SMA; however, only difference in MMP2 and MMP9 were statistically significant. Overall, on day 5, neither TGF-β1 nor CdCl_2_ plus TGF-β1-treated lung slices exhibited significant difference in expressing the genes related with lung fibrosis such as Col 1a1, α-SMA or MMP1, nor did they appear different histology feature in comparison with the untreated lung slices (data not shown).

Ex vivo fibrogenesis was further evaluated with longer cultured time, lung slices were maintained with the presence of TGF-β1 for additional 2 days (day 7) or 5 days (day 10) and then were harvested for MMP1 and MMP7 gene expression analysis. MMP1 gene is considered a biomarker gene of idiopathic pulmonary fibrosis (IPF), the appearance of MMP1 is strongly correlated with IPF in clinic studies. Interestingly, expression of MMP1 in CdCl_2_ plus TGF-β1-treated group was consistently higher than the untreated or TGF-treated groups, although statistical results in each single day did not achieve a *p* value of significant difference. When the data from days 5, 7 and 10 were pooled for analysis, a *p* value of 0.012 was obtained indicating a significant differential expression of MMP1 gene in the lung slices treated with CdCl_2_ plus TGF-β1, but not in the lung slices treated with TGF-β1 (Table 2). As a control, the expression of MMP7 gene was not significantly different from the untreated group (Table 3).

**Table 2:** MMP1 gene expression (fold change)

	Day 5		Day 7		Day 10		Days 5–10	
	Mean	SEM	Mean	SEM	Mean	SEM	Mean	SEM
Untreated	1.00	0.00	1.00	0.00	1.00	–	1.00	0.00
TGF-β1	0.75	0.29	1.48	0.82	0.58	–	0.94	0.35
Cd + TGF-β1	3.56	2.34	2.60	0.42	2.24		2.27	0.76

No observation of statistical significance on individual day, however, a *P* value of 0.012, was obtained when data from day 5, day 7 and day 10 were pooled. Statistical analysis was performed using one sample *T* test, *N* = 4 for day 5, *N* = 5 for day 7 and *N* = 1 for day 10

**Table 3:** MMP7 gene expression (fold change)

	Day 5		Day 7		Day 10		Days 5–10	
	Mean	SEM	Mean	SEM	Mean	SEM	Mean	SEM
Untreated	1.00	0.00	1.00	0.00	1.00	–	1.00	0.00
TGF-β1	0.13	0.46	− 0.03	0.19	− 0.27	–	− 0.02	0.25
Cd + TGF-β1	0.42	0.46	0.30	0.30	− 0.05		0.29	0.31

No observation of statistical significance on individual day nor in pooled data from day 5 to day 10. Statistical analysis was performed using one sample *T* test; *N* = 2 for day 5, *N* = 4 for day 7 and *N* = 1 for day 10

**Fig. 4:**
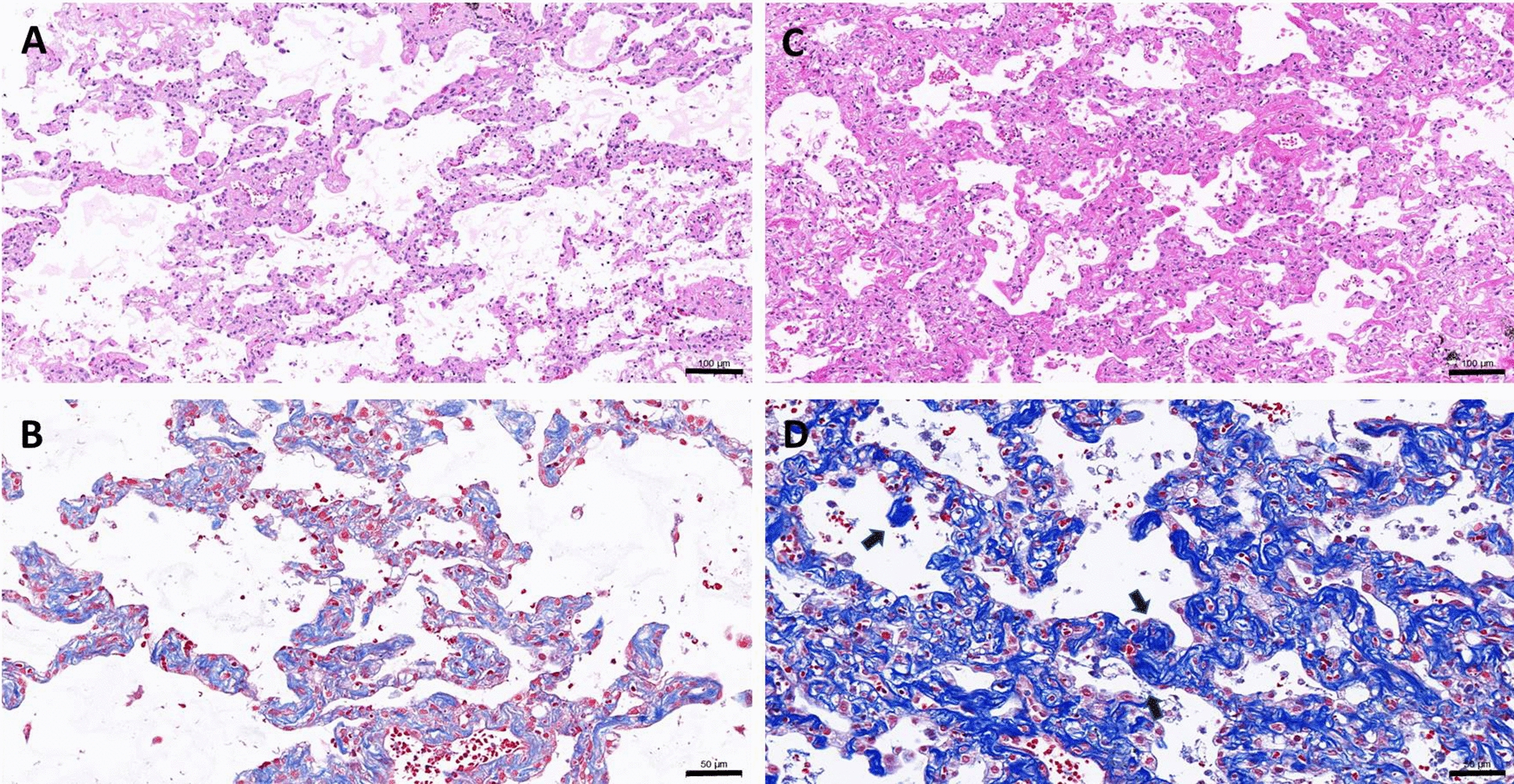
Fibrogenesis of the lung slices cultured for 7 days. Lung slices were treated with TGF-β1 (**a**, **b**) or CdCl_2_ plus TGF-β1 (**c**, **d**) as described in the previous section. They were harvested for histology process and the sections were stained with H/E (**a**, **c**) or Masson’s Trichrome stain (**b**, **d**). Arrows indicate the sphere-like pathogenic features and bars indicates 100 μm (**a**, **c**) and 50 μm (**b**, **d**)

**Fig. 5:**
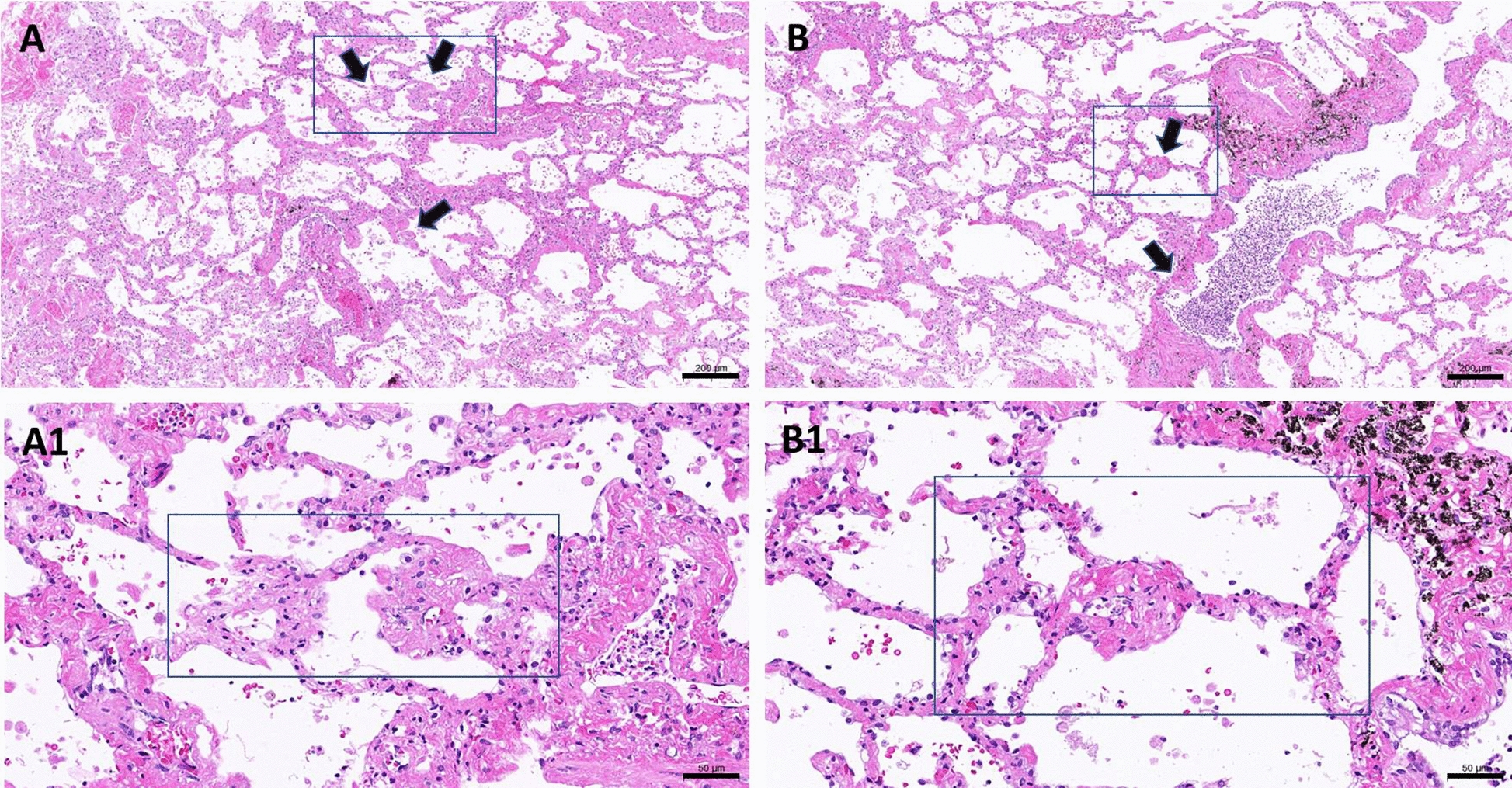
Peri-microvascular fibrous features observed in the lung slices treated with CdCl_2_ plus TGF-β1. Following the treatment as described in the previous treatment, lung slices were further cultured for 3 days and harvested at day 8. Arrows indicate the site of peri-vascular fibrosis observed with lower magnification (**a**, **b**, bar indicate 200 μm); higher magnifications were shown in *a*1 and *b*1, where bars indicate 50 μm

**Fig. 6:**
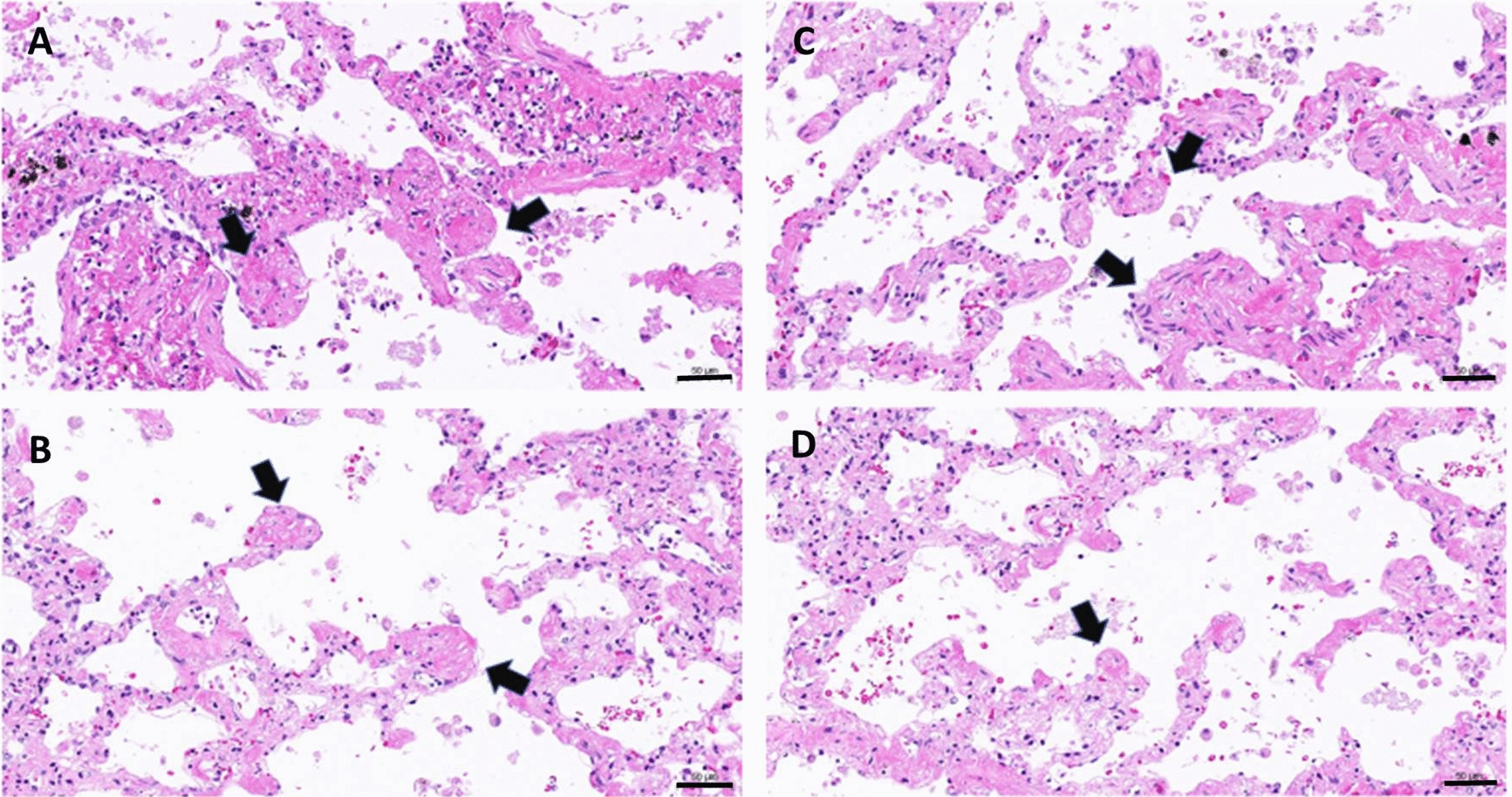
Fibroblast foci-like features observed in the lung slices treated with CdCl_2_ plus TGF-β1. Following the treatment as described in the previous treatment, lung slices were further cultured for 3 days and harvested at day 8. Arrows indicate the fibroblast foci-like pathogenesis observed in different lung slices obtained from two independent experiments (**a**, **b**; **c**, **d**). Bars indicate 50 μm

Histology observations on the day 7 lung slices further revealed clear fibrous features demonstrated by thickened alveolar septa (Fig. 4a, c) with excess existence of collagen fibers (Fig. 4b, d) in the lung slices treated with TGF-β1 (A and B) and CdCl_2_ plus TGF-β1 (C and D). It is noteworthy that the in CdCl_2_ plus TGF-β1-treated lung slices seemed to differ from the TGF-β1-treated ones in the appearance of sphere-like features (Fig. 4d, arrow) and thicker alveolar septa. More observations over the day 8 harvested lung slices treated with CdCl_2_ plus TGF-β1 further confirmed the existence of such features which can be peri-microvascular (Fig. 5) or fibroblast foci-like (Fig. 6), which the adjacent alveolar septa remain a diffusion fibrosis feature.

## Discussion

The use of human lung slices in translation research has recently attracted great interest, where, in most of the cases, lung slices were prepared from an intact lung lobe. To facilitate the application of ex vivo model, we investigate the feasibility of preparing lung slices from small pieces of surgery biopsy. Such preparation method is validated from the perspectives of cadmium-induced cytotoxic and gene expression profile against the results obtained from rodent model. In addition, its suitability for pathological approaches is examined by treating lung slices with CdCl_2_ plus TGF-β1 which was previously reported successful in ex vivo fibrogenesis.

Cadmium exposure for 3 days resulted in a dose-dependent toxicity in the both human and rat lung slices. In consistent with the previous findings with cultured cells [11], human lung slices exhibited a stronger resistance to cadmium challenge in comparison with rat lung slices. Therefore, the use of reported human lung slice for toxicology assessment is validated by the result of cadmium-induced differential toxicity profile. Histology results also reveal a dose-related interstitial damages in the human lungs slices following 3 days of cadmium treatment. Interestingly, an emphysematous-like alveolar space enlargement with interstitial fibrosis was observed in the lung slices treated with cadmium at 40 and 80 μM, the latter is accountable for a 50% relative toxicity. The lungs of animals exposed to cadmium developed fibrosis and emphysema [18, 20, 23]. It is not clear, in our model, why a 3-day cadmium exposure could result in the concurrence of fibrosis and alveolar enlargement; yet, our results demonstrate an active lung repair activity remains in the lung slices with or without the presence of cadmium chloride.

Since ECM imbalance underlines the pathogenesis of interstitial lung diseases including fibrosis and emphysema [24, 25], the expression of ECM metabolism genes in current model was further examined. Following 3 days of cadmium treatment at 30 μM, MMP2 and MMP9 genes expression were significantly higher in comparison with the untreated group, but not α-SMA, Col1a1, MMP1 or MMP7. These results are consistent with the previous findings that cadmium-induced MMP2 and MMP9 expression in rat lungs [19]. Type IV collagen is the main collagen of interstitial lung architecture as well as a substrate of MMP2 and MMP9 [26]. It is highly possible that MMP2 and MMP9 activity play a role in alveolar deterioration along with the cadmium-induced cytotoxicity at higher concentration. Increasing in myofibroblast cell number is a key feature of lung fibrosis [27, 28]; however, we do not observe a significant increase in the expression of α-SMA and collagen 1a1 gene which are the translational biomarkers of myofibroblast, respectively [29, 30]. Cadmium treatment for 3 days at 30 μM resulted in approximate 25% cytotoxicity which may be the appropriate for MMP2 and MMP9 gene induction but not potent enough for the significant induction of α-SMA and collagen 1a1 gene expression.

Appearance of diffuse alveolar fibrosis in the untreated was noted (Fig. 3a). Lung slices prepared through such intensive manual processes will inevitably impose certain physical stress and consequently generate damages to the tissues before commence of ex vivo culture in the incubator. It is not surprising that the untreated lung slices can develop fibrosis along the time of culture. This was then followed at the day of preparation (day 0), at the end of acclimation (day 2), and at the day of harvesting (day 5). Histological observations in Fig. 1 clearly demonstrate the formation of diffuse alveolar fibrosis over time and ECM, indicated by trichrome stain, is accountable for the increasing thickness of the alveolar wall. With this in mind, all analytical data generated in relative to the untreated group should be interpreted with the concern of intrinsic fibrogenesis in the untreated lung slices. Thus, insignificant induction of α-SMA and collagen 1a1 gene expression in the 30 μM cadmium-treated lung slices can be partially explained.

Current working hypothesis for the lung fibrosis is that repeated lung damage couples with impaired tissue repair process chronically results in accumulation of ECM and some cytokines such as TGF-β1 and CTGF potentiate the impaired repair process [31]. This hypothesis is supported by rodent ex vivo experimental models where increasing thickness of alveolar septa (14-day culture) and early signs of fibrosis (3-day culture) were reported by cadmium plus TGF-β1 treatment [16, 32]. To evaluate the applicable feasibility of current human lung slice preparation in ex vivo fibrogenesis, similar approach was then taken with the co-treatment of cadmium (30 μM) with TGF-β1 (1 ng/ml). Note that TGF-β1 (1 ng/ml) was determined as it alone does not induce higher expression of α-SMA, or collagen 1a1 or MMP1 gene. Interestingly, MMP2 and MMP9 gene expression were increased which is in consistent with previous finding indicating the similar effect of TGF-β1 to cultured cell lines [33–35]. Following 3 days of co-treatment, none of the gene expression examined was found significantly increased in comparison with the untreated group (Table 1) indicating a 3-day co-treatment is inadequate to push the progress of fibrosis further than that in control lung slices.

In this co-treatment protocol, cadmium is an agent for damaging tissue and to activate the repair process, while TGF-β1 is expected to potentiate the impair process. Therefore, longer period of culturing time was taken without the presence of cadmium but TGF-β1 remains until the day of harvesting. Very interestingly, expression of MMP1 was found increasing at every harvest timepoint and statistical results gave a *p* value slightly higher but very close to 0.05. We, therefore, pooled the data together from days 5, 7, and 10 to increase the sample size and a *p* value less than 0.05 was obtained (Table 2), while MMP7 gene induction is not apparent (Table 3). Both MMP1 and MMP7 are found significantly higher in the lung and blood of patients with IPF [36, 37], the latter was considered specifically for the biomarker of disease progress [38]. Such differential expression pattern in the current lung slice model might be the outcome of early stage of lung fibrosis or the lung slice sampled from the lung apices rather than from the bases [39]. The potentiation power of TGF-β1 is revealed in Fig. 4 that the co-treatment group appears with thicker alveolar septa showing excess ECM than that of TGF-β1 group. In addition, some ECM contented sphere-like structures were observed (Fig. 4d) in the co-treated lung slices and that repeatedly appear on the day 8 samples collected from different independent experiments. These sphere-like features are either peri-microvascular (Fig. 5) or early stage fibroblast foci (Fig. 6). Increased MMP1 gene expressions as well as the appearance of fibroblast foci-like structure strongly suggest a pathogenesis of early IPF.

Applying small lung tissues instead of whole lung lobe for slice preparation is a valid approach considering limited human lung availability; improvement is required to reduce the physical stress imposed throughout the preparation process. Cadmium-induced response as well as the results of co-treatment protocol provide results consistent with the published data and thus validate the current model as a potential tool for lung fibrosis translation research, noting that the treatment regimen may be adjusted for ideal or better outcomes.

## Conclusion

Our results show that interstitial lung fibrosis can be the consequence of tissue damage plus impaired repair process in the presence of limited blood-borne inflammatory responses, since the lung slice culture is in short of blood supply and the inflammatory response is considered minimum.

## Data Availability

Not applicable.
